# Incidence and severity of electric scooter related injuries after introduction of an urban rental programme in Vienna: a retrospective multicentre study

**DOI:** 10.1007/s00402-020-03589-y

**Published:** 2020-08-27

**Authors:** Timon Moftakhar, Michael Wanzel, Alexander Vojcsik, Franz Kralinger, Mehdi Mousavi, Stefan Hajdu, Silke Aldrian, Julia Starlinger

**Affiliations:** 1 Department of Orthopaedics and Trauma-Surgery, Medical University of Vienna, Vienna, Austria; 2 Department of Trauma Surgery, Wilhelminenspital, Vienna, Austria; 3 Department of Trauma Surgery, Sozialmedizinisches Zentrum Ost Donauspital, Vienna, Austria; 4 Austrian Cluster for Tissue Regeneration, Vienna, Austria; 5 Department of Orthopedic Surgery, Mayo Clinic Rochester, Rochester, MN USA

**Keywords:** Electric scooter, Scooter share, Trend sport, Injury pattern, Injury severity, Injury prevention, Trauma, Fracture, Head injury, Emergency department, Epidemiology, Retrospective study

## Abstract

**Purpose:**

Electric scooters (e-scooters) are an emerging way of mobility in cities around the world. Despite quickly rising numbers of e-scooters, limited studies report on incidence and severity of e-scooter-associated injuries. The aim of our study was to report on these injuries and identify potential protective measures to ultimately decrease e-scooter-associated morbidity.

**Methods:**

We performed a retrospective multicentre study including all patients, who were admitted to three major trauma departments in Vienna from May 2018 to September 2019. We analysed patients’ data, including demographics, injury pattern, types of injury and subsequent treatment.

**Results:**

A total number of 175 patients (115 males, 60 females) sustained e-scooter-associated injuries. Patients’ mean age was 34.4 years [4–74]. While the mean Injury Severity Score (ISS) was 3.4, 11 patients presented with an ISS ≥ 9 and 2 patients with an ISS ≥ 16. ISS increased with age. Older patients (≥ 40 years) presented a significantly higher ISS than younger patients (< 40 years) (*P* = 0.011). Seventy-one patients (40.6%) sustained major injuries affecting head (35.2%) and upper extremities (36.6%). Twenty-three patients (13.1%) required surgery leading to hospitalization of 11 days on average [1–115]. E-scooter-associated injuries increased during late afternoon plateauing at 8.00 pm. However, the largest share of patients (39.2%) sustained their injuries during early night (8.00 pm to 1.59 am) with especially young adults (19–39 years) being at risk.

**Conclusion:**

The popularity of rideshare e-scooters across cities worldwide seems to be on the rise, so are e-scooter-associated injuries. These injuries should be considered high-energy trauma affecting primarily head and upper extremity; indeed, 17.7% sustained major head injuries. Therefore, the mandatory use of a helmet seems to be adequate to decrease head injury-associated morbidity. Ultimately, given the remarkably high rates of nighttime injuries, an e-scooter ban during night could further cut injury numbers in half.

## Introduction

Across city centres worldwide, electric scooters (e-scooters) have become a popular means of transportation. In Vienna, Austria, commercial providers increased the number of available e-scooters up to about 6000 in April 2019. Doing so, the number of e-scooter-associated injuries in trauma departments across town continued to go up [1].

Recently, several authors reported on this trend [2, 3], e.g. Bekhit et al. [2] observed a striking increase of e-scooter-associated injuries in Auckland, New Zealand, from 2 up to 35 injuries per week after the introduction of an urban e-scooter sharing system. Namiri et al. [4] did an extensive analysis utilizing the National Electronic Injury Surveillance System reporting a significant increase of e-scooter-associated injuries in the USA from 4582 in 2014 to 14,651 in 2018.

In addition to the rising number of e-scooter-associated injuries, the severity of reported injuries is considerable, given the fact that riders on the street typically use speeds up to 20 miles/h corresponding to 32 km/h. For instance, Badeau et al. [3] report on 44% major injuries in e-scooter-associated emergency room (ER) admissions with 14% of all patients requiring surgery. Hence, e-scooter-associated injuries are beyond negligible occasionally resulting in fatal accidents.

The severity of injuries reflects the burden for the individual patient. Moreover, the injury severity together with the increasing incidence raises concerns about costs due to e-scooter-related accidents. In a study period of 7 months, e-scooter-associated injuries caused a total amount of $1.3 million New Zealand dollars (NZD), resulting in an estimated cost of $1300 NZD per rentable e-scooter as reported by Bekhit et al. [2]. Similarly, Campbell et al. [5] report on 23 operations which were necessary due to e-scooter-associated injuries. Doing so, the authors estimate a total economic cost of $404,925 NZD caused by this subset of e-scooter-associated injuries.

Ridesharing e-scooters is of emerging popularity, but this trend does not come without consequences as outlined in the most recent literature. In the participating trauma departments, an increasing number of injuries due to accidents involving e-scooters have been observed. Thus, the purpose of our study was to investigate the incidence, severity and a potential injury pattern of e-scooter-associated injuries in an effort to ultimately increase user’s safety, e.g. by mandatory helmet use or advanced lighting technologies [2–4, 14].

## Materials and methods

We performed a retrospective multicentre study including all consecutive patients who were admitted for an e-scooter-associated injury (rider as well as non-rider) from May 1, 2018 to September 30, 2019. We therefore collected data from three of Vienna’s major trauma departments: “Vienna General Hospital—Medical University of Vienna”, “Sozialmedizinisches Zentrum Ost—Donauspital” and “Wilhelminenspital Wien”).

The study was approved by the Institutional Review Board (IRB) (Ethics Committee of the Medical University of Vienna, No. 1804/2019) as well as the Ethics Committee of the City of Vienna (No. 19-213-VK) and conducted in accordance with the declaration of Helsinki. Due to the retrospective nature of the cohort study, no informed consent was required by the IRB. All medical records were reviewed for eligibility (e-scooter-associated injury) and data were collected by scanning patients’ medical histories, outpatient documentations, surgery reports and discharge summaries (date and time of injury, age, sex, type of introduction, mechanism of injury, influence of alcohol, type of injuries, treatment, duration of hospitalization and duration of outpatient aftercare). After data collection and prior to statistical workup, patients’ records were anonymized.

All injuries were classified according to the Injury Severity Score [15]. Accordingly, we categorized patients presenting with an ISS ≥ 9 as moderately injured. Moreover, we classified all injuries into “major” (fractures, dislocations, concussions, intracranial haemorrhages) and “minor” injuries (contusions, soft tissue injuries, strains or sprains). Further, we categorized injury locations into head, upper extremities including shoulder girdle, lower extremities including pelvis and thorax including spine.

### Statistical analysis

Statistical analysis was performed using SPSS 26.0 software (SPSS Inc., Chicago, IL). Comparisons between independent groups of continuous variables were performed by independent sample *t* test and Chi-square test. The statistical significance level was set to *P* ≤ 0.05.

## Results

### A noticeable increase of e-scooter-associated injuries was observed from 2018 to 2019

**Fig. 1:**
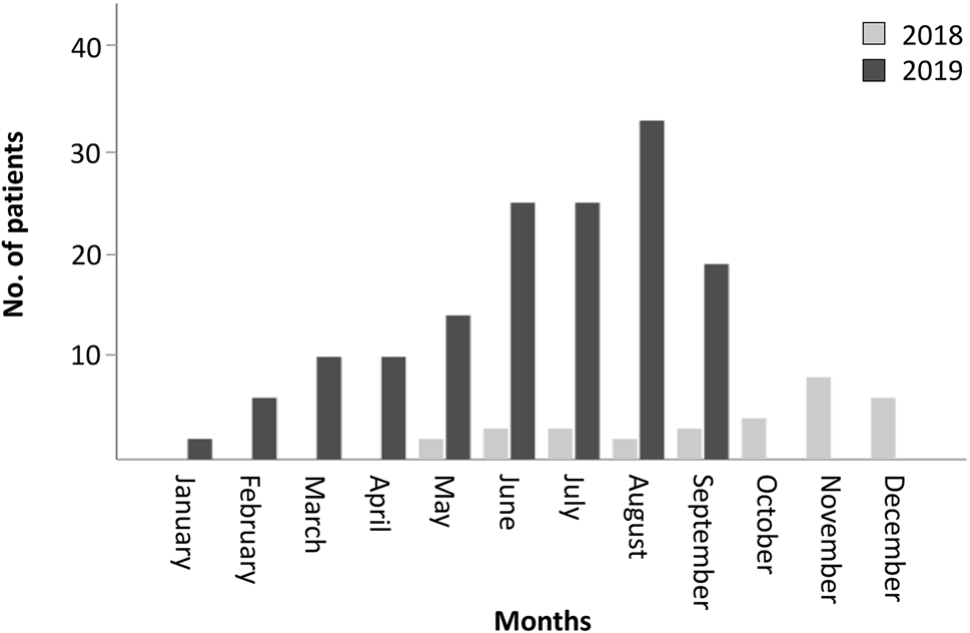
Bargraph illustrating the increase of e-scooter-associated injuries in Vienna from 2018 to 2019 after the implementation of e-scooter sharing programmes

We observed a distinct increase of e-scooter-associated injuries from 2018 to 2019 resulting in a total number of 175 patients with e-scooter-associated injuries during the study period. During the overlapping 5-month summer period (May–September), we observed a considerable increase of e-scooter injuries from 2018 (*n* = 13) to 2019 (*n* = 116), representing an 892% increase (Fig. 1). Patients involved in e-scooter accidents were predominantly male (65.7%) with a mean age of 34.4 years, ranging from 4 to 74 years. Details on patients’ characteristics are shown in Table 1.

**Table 1:** Patients’ demographics

	*n* (%)
Age in years (mean [range])	34.4 [4–74]
Sex	
Male	115 (65.7)
Female	60 (34.4)
Mode of arrival	
Ambulance	60 (34.3)
Walk in	99 (56.6)
n.a.	16 (9.1)
Time of injury	
Daytime (8.00 am to 7.59 pm)	64 (38.6)
Night-time (8.00 pm to 7.59 am)	102 (58.3)
n.a.	9 (5.1)
Treatment	
Outpatient treatment	125 (71.4)
Hospital admission	47 (26.9)
Not documented	3 (1.7)
Length of hospital stay in days (mean [range])	6.5 [1–115]
Type of treatment	
Non-operative management	152 (86.9)
Operative management	23 (13.1)
Aftercare	
Outpatient aftercare	59 (33.7)
Length of outpatient aftercare in days (mean [range])	38.9 [1–368]
Mechanism of injury	
Rider	166 (94.9)
Non-rider	9 (5.1)

### E-scooter-associated injuries affected head, upper and lower extremity to the same extent

**Fig. 2:**
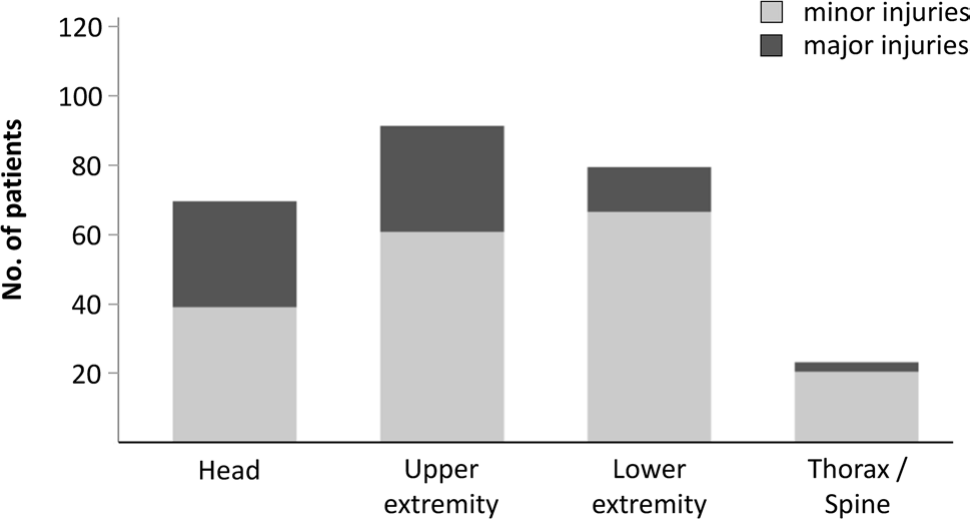
Distribution of injuries in terms of anatomic location and severity

A total of 104 patients (59.4%) presented with minor injuries, whereas 71 patients (40.6%) presented with major injuries: of those 71 patients with major injuries, 6 patients (8.5%) presented with major injuries in more than one region. With respect to the anatomic location, we could not identify a typical injury pattern: 25 patients (35.2%) sustained a major head injury, 26 patients (36.6%) a major injury of the upper extremity, 12 patients (16.9%) a major injury of the lower extremity, and 2 patients (2.8%) a major injury of thorax or spine (Fig. 2). Further details on injury characteristics are listed in Table 2.

**Table 2:** E-scooter-associated injuries with respect to the anatomic location

	*n*
Head injuries (patients)	71
Fractures	21
Facial bones	18
Base of the skull	3
Intracranial haemorrhages	7
Subdural	3
Subarachnoid	3
Epidural	1
Concussions	21
Contusions of the skull	55
Fractures (non-head) (patients)	44
Upper extremities including shoulder girdle	33
Lower extremities including pelvis	15
Thorax or spine	4
Dislocations (patients)	2
Shoulder joint	2
Contusions (patients)	82
Strains or sprains (patients)	20
Soft tissue injuries (abrasions, lacerations or haematomas) (patients)	108

Categories are not mutually exclusive

### The majority of e-scooter-associated injuries were managed non-operatively

From 175 patients, 47 were admitted to the hospital. About half of them (23 patients, 14 males, 9 females) required operative management (OM) and underwent a total of 27 surgical procedures. The average age of patients who underwent OM was 34.1 years [13–63]. Their ISS was significantly higher (6.8 [1–27]) compared to those patients who underwent non-operative management (NOM) (2.9 [1–14], *P* = 0.007). Patients treated with OM tended to have increasing length of hospital stay compared to those with NOM (OM 11.0 days [1–115], NOM 3.0 days [1–19], *P* = 0.177). Outpatient aftercare was significantly longer in patients who underwent OM compared to patients receiving NOM (OM 105.3 days [7–368], NOM 16.3 days [1–67], *P* = 0.006). The most common surgeries were open reduction and internal fixation of fractures. Different types of surgeries performed are shown in Table 3.

**Table 3:** Types of surgeries

	*n*
Osteosynthesis	17
Internal fixation with screws and/or plates	13
Percutaneous pinning	1
Tension band wiring	1
Intramedullary fixation	2
Neurosurgical interventions	3
Maxillofacial surgery	3
Ear, nose and throat (ENT) surgical procedure	1
Interventional radiological surgery	1
Soft tissue surgery	2

### Severity of e-scooter-associated injuries increased with age

**Fig. 3:**
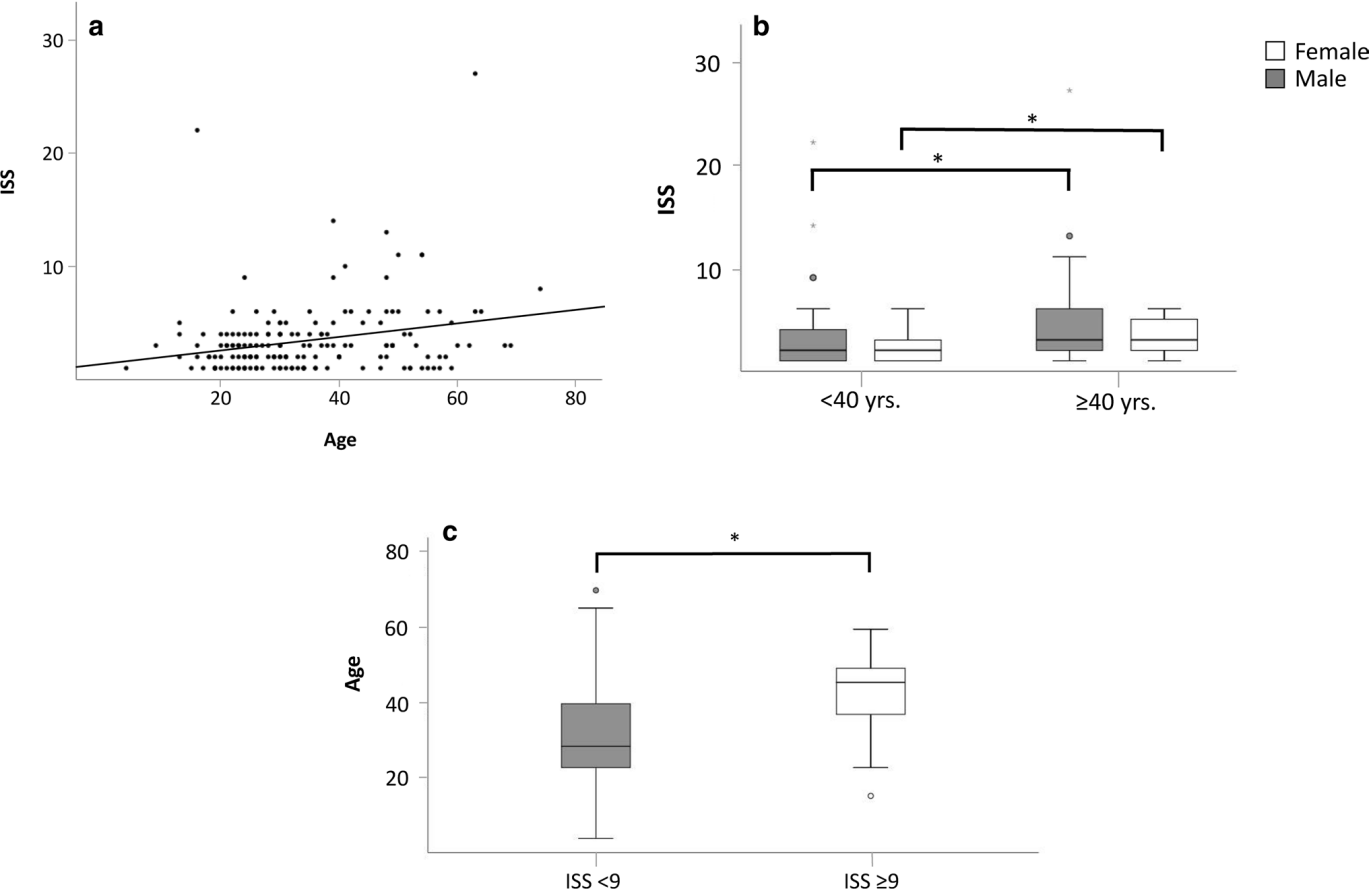
**a** Scatterplot illustrating the significant relationship between age and ISS (*P* < 0.001, *R* = 0.325). **b** Box plot showing the significantly higher ISS Scores of adults ≥ 40 years compared to patients < 40 years irrespective of sex. *Significant difference (*P* ≤ 0.05). **c** Box plot showing that patients with an ISS ≥ 9 were significantly older than patients ISS < 9 (*P* = 0.027). *Significant difference (*P* ≤ 0.05)

We observed a noticeable trend indicating that ISS was age dependent (*P* = 0.001, *R* = 0.248) as visualized in Fig. 3a. This difference was even more pronounced classifying patients into three age groups: adolescents (≤ 18 years, *n* = 13), young adults (19–39 years, *n* = 107) and older adults (≥ 40 years, *n* = 55). Especially patients ≥ 40 years sustained more severe injuries, with respect to their ISS; irrespective of sex, this difference was found to be significant (ISS in all patients:  < 40 years 2.9 [1–22],  ≥ 40 years 4.6 [1–27], *P* = 0.011; ISS in females: < 40 years 2.5 [1–6],  ≥ 40 years 3.4 [1–6], *P* = 0.042; ISS in males:  < 40 years 3.1 [1–22],  ≥ 40 years 5.1 [1–27], *P* = 0.032; Fig. 3b).

Looking at a clinically relevant cutoff of ISS ≥ 9, this relationship was found to be even more pronounced. Doing so, we found patients with an ISS ≥ 9 to be significantly older than patients with an ISS < 9 (ISS ≥ 9 43.3 years [16–63], ISS < 9 33.8 years [4–74], *P* = 0.010; Fig. 3c).

### E-scooter-associated injuries increased in the afternoon and plateaued during the night

**Fig. 4:**
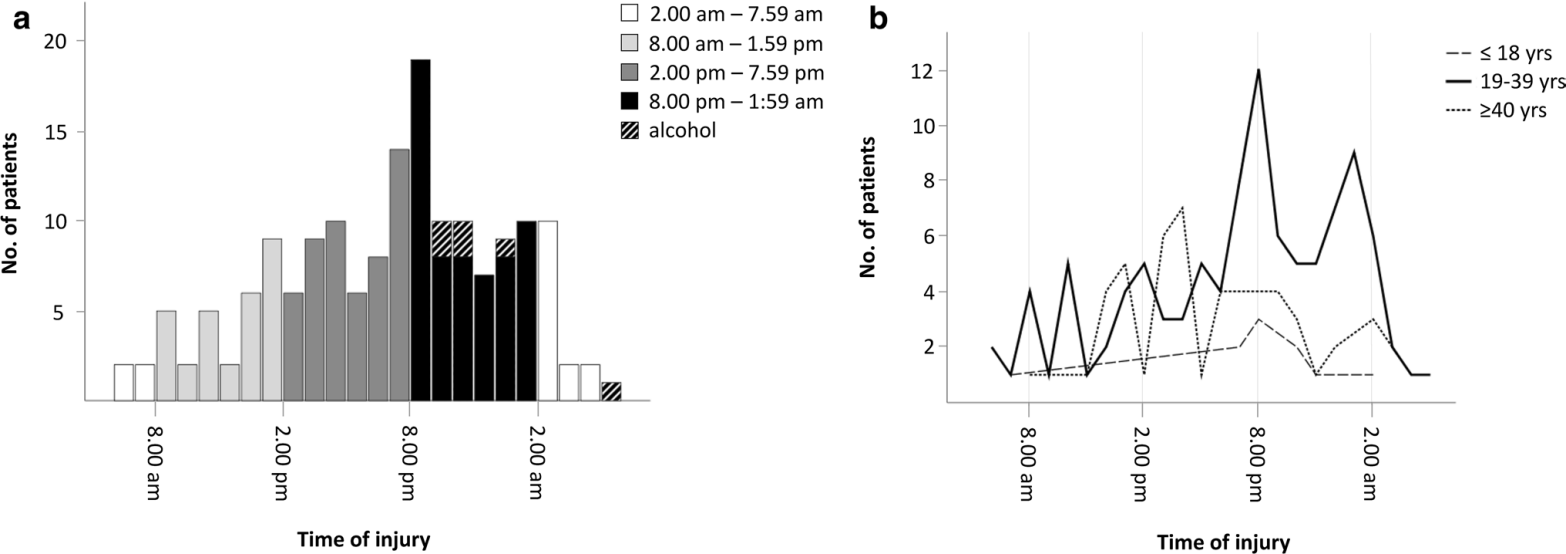
**a** Distribution of the rate of injuries in respect of the time of injury. **b** Line chart illustrating the different rates of injuries of the three classified age groups (adolescents, young adults and older adults) over the course of the day

Eighty-two patients (49.4%) presented during daytime (8.00 am to 7.59 pm), while 84 patients (50.6%) sustained their injury during nighttime (8.00 pm to 7.59 am). A subgroup analysis dividing the two groups into morning and afternoon as well as early night and late night revealed a distinct increase of e-scooter-associated injuries during the afternoon plateauing at 8.00 pm. Ultimately, the largest share of patients (39.2%) sustained the injuries during early night (8.00 pm to 1.59 am) (Fig. 4a). With respect to the patients’ age, we found older adults (≥ 40 years) to be more likely to fall in the afternoon compared to young adults (19–39 years) who were at highest risk to fall during early nighttime (Fig. 4b). Out of all patients, seven patients (4.0%) stated to have been under the influence of alcohol at the time of the injury, and all alcohol-associated injuries happened after 8 pm.

## Discussion

The popularity of e-scooters is continuously increasing, so is the number of e-scooter-associated injuries. In our study, we observed a noticeable rise of e-scooter-associated ER admissions from 13 in summer 2018 (May–September) to 116 in the respective period in 2019. This corresponds to an increase of 892% of e-scooter-associated injuries in Vienna, Austria.

While the severity of injuries varied in our patients (mean ISS 3.4 [1–27]), 40.6% sustained a major injury (fracture, dislocation, intracranial haemorrhage or concussion). Eleven patients presented with an ISS ≥ 9 and 2 further patients with an ISS ≥ 16. Interestingly, we found a considerable increase of ISS with age irrespective of sex. Indeed, patients presenting with an ISS ≥ 9 were significantly older than less severely injured patients. This emphasizes the fact that e-scooter-associated injuries are beyond benign and should be considered as high-energy trauma injuries with potentially life-threatening extent.

With respect to the anatomic area, we found the highest number of e-scooter-associated injuries for the upper extremity (53.1%). As similar trend sports (e.g. riding a micro-scooter or roller-skating) typically cause fractures of the wrist region, the authors usually recommended wrist protectors for the prevention of these distinct injury patterns [6, 7]; so did Ishmael et al. [8] by reporting on e-scooter-associated injuries requiring surgery, as 11 out of 75 patients sustained wrist fractures. Interestingly, we did not find a typical injury pattern for e-scooter-associated injuries affecting the wrist. Indeed, elbow fractures were more likely than wrist fractures (11 versus 3) in our patients. While Ishmael et al. [8] recommended the use of wrist guards for e-scooter riders, based on our numbers, the overall benefit might be only marginal.

The use of protective gear is worthwhile considering, given the high number of head injuries observed in our patients: we found the head to be at major risk for e-scooter-associated injuries, as 40.6% of the patients sustained an injury in this region with 43.7% sustaining a major head injury. These findings go in line with the report by Trivedi et al., who had observed 40.2% head injuries studying 249 e-scooter-associated injuries [9].

At the same time, the city of Brisbane passed a law for compulsory helmet use for e-scooter riders in January 2019. Interestingly, Mitchell et al. studied the incidence of e-scooter-associated injuries in Brisbane from November 2018 to January 2019, covering the time when the use of helmets became mandatory. Doing so, the related effect was considerable as the authors observed a subsequent helmet use in 46% of their patients. The authors further concluded that the use of a helmet at the time of injury significantly decreased the risk of sustaining a head injury compared to patients not wearing a helmet [10]. This goes in line with reports studying the protective potential of helmets in other road users. Olivier et al. [11] presented similar findings reporting that helmet use while riding a bicycle is associated with a significant reduction of head injuries, corresponding to an odds ratio of 0.49 by the use of a helmet. Namiri et al. reported more than one-third of all e-scooter-associated injuries affecting the head region, leading to more than double the rate of head injuries, related to bicycle injuries [4, 12].

Currently available data on head injuries in e-scooter riders report heterogeneous numbers being as high as up to 40.2% [3, 9, 13]. Unlike in Brisbane, Australia, the use of a helmet is not mandatory for riding an e-scooter in any European capital. To this end, a law for compulsory use of helmet for e-scooter riders could lead to a significant reduction of e-scooter-associated head injuries and seems of utmost importance.

Several authors have already reported increased numbers of ER admissions during the evening and nighttime [9, 14]. Doing so, Trivedi et al. [9] reported that 56.6% of e-scooter-associated injuries occurred between 3.00 pm and 11.00 pm. Similarly, Blomberg [14] found that 68 patients (52.3%) sustained their injury between 3.00 pm and 11.00 pm and 38 patients (29.2%) were injured between 11.00 pm and 7.00 am. Our results are in line with the above-mentioned observations, as we also observed a continuous increase in the evening with a peak around 8.00 pm. However, most of the injuries in our study occurred during night (8.00 pm to 7.59 am) affecting primarily young adults. Given this pronounced peak during the night, an e-scooter ban at night might substantially decrease the number of injuries and should therefore be taken into account. Alternatively, advanced built-in lighting technologies to enhance the visibility of e-scooter riders could lead to a reduction of e-scooter-associated accidents during nighttime.

Moreover, seven patients self-reported to be riding the e-scooter under the influence of alcohol at the time of the injury. All seven patients sustained major head injuries, including one patient with an additional major injury of the upper extremity. Thus, e-scooter riders under the influence of alcohol had a significantly higher risk of sustaining major injuries (*P* = 0.001). Given the fact that these seven patients were injured during the nighttime, a possible night ban for e-scooters could likewise reduce the number of injuries sustained under the influence of alcohol.

## Conclusion

Given the rising numbers of e-scooter-associated injuries seen in trauma departments across Vienna, this study aims to report on the severity and injury pattern of this new means of transportation. In addition to the currently available literature, we report on a high number of patients sustaining head injuries [3, 4, 9, 13]. Thus, the mandatory use of a helmet seems an adequate measure to prevent these injuries in the future. Moreover, banning the use of e-scooters during the overnight hours should be evaluated as more than 50% of e-scooter-associated injuries happened during nighttime in our patients as well as in several other reports [9, 14]. To this end, e-scooter riders are the least protected road users, therefore it is necessary to implement extended safety precautions.
